# Continuous production of biohythane from hydrothermal liquefied cornstalk biomass via two-stage high-rate anaerobic reactors

**DOI:** 10.1186/s13068-016-0666-z

**Published:** 2016-11-21

**Authors:** Bu-Chun Si, Jia-Ming Li, Zhang-Bing Zhu, Yuan-Hui Zhang, Jian-Wen Lu, Rui-Xia Shen, Chong Zhang, Xin-Hui Xing, Zhidan Liu

**Affiliations:** 1 Laboratory of Environment-Enhancing Energy (E2E), and Key Laboratory of Agricultural Engineering in Structure and Environment, Ministry of Agriculture, College of Water Resources and Civil Engineering, China Agricultural University, Beijing, 100083 China; 2 Department of Agricultural and Biological Engineering, University of Illinois at Urbana-Champaign, Urbana, 61801 USA; 3 Key Laboratory of Industrial Biocatalysis of Ministry of Education of China, Beijing, 100084 China; 4 Department of Chemical Engineering, Institute of Biochemical Engineering, Tsinghua University, Beijing, 100084 China

**Keywords:** Biohythane production, Hydrothermal liquefaction, Biomass, Two-stage fermentation, Microbial community

## Abstract

**Background:**

Biohythane production via two-stage fermentation is a promising direction for sustainable energy recovery from lignocellulosic biomass. However, the utilization of lignocellulosic biomass suffers from specific natural recalcitrance. Hydrothermal liquefaction (HTL) is an emerging technology for the liquefaction of biomass, but there are still several challenges for the coupling of HTL and two-stage fermentation. One particular challenge is the limited efficiency of fermentation reactors at a high solid content of the treated feedstock. Another is the conversion of potential inhibitors during fermentation. Here, we report a novel strategy for the continuous production of biohythane from cornstalk through the integration of HTL and two-stage fermentation. Cornstalk was converted to solid and liquid via HTL, and the resulting liquid could be subsequently fed into the two-stage fermentation systems. The systems consisted of two typical high-rate reactors: an upflow anaerobic sludge blanket (UASB) and a packed bed reactor (PBR). The liquid could be efficiently converted into biohythane via the UASB and PBR with a high density of microbes at a high organic loading rate.

**Results:**

Biohydrogen production decreased from 2.34 L/L/day in UASB (1.01 L/L/day in PBR) to 0 L/L/day as the organic loading rate (OLR) of the HTL liquid products increased to 16 g/L/day. The methane production rate achieved a value of 2.53 (UASB) and 2.54 L/L/day (PBR), respectively. The energy and carbon recovery of the integrated HTL and biohythane fermentation system reached up to 79.0 and 67.7%, respectively. The fermentation inhibitors, i.e., 5-hydroxymethyl furfural (41.4–41.9% of the initial quantity detected) and furfural (74.7–85.0% of the initial quantity detected), were degraded during hydrogen fermentation. Compared with single-stage fermentation, the methane process during two-stage fermentation had a more efficient methane production rate, acetogenesis, and COD removal. The microbial distribution via Illumina MiSeq sequencing clarified that the biohydrogen process in the two-stage systems functioned not only for biohydrogen production, but also for the degradation of potential inhibitors. The higher distribution of the detoxification family *Clostridiaceae*, *Bacillaceae*, and *Pseudomonadaceae* was found in the biohydrogen process. In addition, a higher distribution of acetate-oxidizing bacteria (*Spirochaetaceae*) was observed in the biomethane process of the two-stage systems, revealing improved acetogenesis accompanied with an efficient conversion of acetate.

**Conclusions:**

Biohythane production could be a promising process for the recovery of energy and degradation of organic compounds from hydrothermal liquefied biomass. The two-stage process not only contributed to the improved quality of the gas fuels but also strengthened the biotransformation process, which resulted from the function of detoxification during biohydrogen production and enhanced acetogenesis during biomethane production.

**Electronic supplementary material:**

The online version of this article (doi:10.1186/s13068-016-0666-z) contains supplementary material, which is available to authorized users.

## Background

Hythane is regarded as a clean and efficient energy as it combines the advantages of both hydrogen and methane [1]. Hythane production using biomass via two-stage anaerobic fermentation is respected as a promising direction [2]. Compared with conventional methane fermentation, the biohythane production process improves energy recovery, reduces fermentation time, and leads to a better control of the microbial community due to the separation of the biohydrogen and biomethane processes [1–3].

Approximately 200 billion tons of lignocellulosic biomass is annually produced worldwide [4]. The main components of lignocellulosic biomass are hemicellulose and cellulose, primarily consisting of C5 and C6 sugars, which could be used for the production of fuels and chemicals [5]. Biohythane production using lignocellulosic biomass has been intensely investigated [3, 6, 7]. However, a long fermentation time and low gas production are observed when using lignocellulose biomass for fermentation [4, 6, 7]. This is mainly due to the natural recalcitrance of the lignocellulosic structure, which makes it difficult to directly and effectively use [4]. Different kinds of pretreatments have been used to break down the structure of lignocellulosic biomass in order to make it less recalcitrant, including mechanical [8], chemical (alkaline, acid) [9, 10], biological [11], hydrothermal methods [12], or a combination of the preceding methods.

Among these, hydrothermal liquefaction (HTL) is a promising technology for the treatment and liquefaction of various biomass sources in which the water itself is an environmentally friendly solvent and reactant [13]. The integration of HTL and anaerobic fermentation has been reported as a way that could enhance methane production from various lignocellulosic biomasses, including sunflower oil cake [14], sorghum forage [15], wheat straw [15, 16], sugar beet [17], rice straws [18], and sunflower stalks [19]. The methane production was increased in a range of 6.5–222% in these studies [14–19]. However, there are still several challenges for successful integration. Firstly, the limited efficiency of fermentation reactors is a large bottleneck, as low HTL temperatures (100–200 °C) [14–19] in these studies resulted in a high solid content feedstock. Usually, batch reactors or conventional continuous stirred anaerobic reactors (CSTR) are used. However, these reactors are well known for their low efficiency, long retention time, and low organic loading rate. Another challenge is the inhibition of fermentation due to toxic organic compounds released during HTL. Furfural (0.08–13.32 g/L), 5-hydroxymethyl furfural (5-HMF) (0.032–4.3 g/L), and phenols (0.15–7.21 g/L) are produced during thermochemical treatment [20]. A number of studies reported that these inhibitive compounds could be degraded during fermentation [21, 22]. However, contradictory results have also been mentioned, and biohydrogen production was suppressed by these inhibitors [20]. Therefore, the degradation of these inhibitors during fermentation, especially two-stage biohythane systems, needs to be specifically investigated.

In this study, a novel strategy for the continuous production of biohythane from cornstalk through the integration of HTL and two-stage fermentation is proposed. HTL was conducted to get a high yield of liquid products from cornstalk. Our recent study [23] reported a high rate of liquefaction (up to 57.89%) and recovery of sugars and volatile fatty acids (VFAs) (up to 92.39% of aqueous products) from cornstalk via HTL. The liquid products from cornstalk after HTL were fed into the fermentation systems. The upflow anaerobic sludge blanket (UASB) and packed bed reactor (PBR) were used to build up the fermentation systems. A high density of microbes was developed in the bioreactors, further leading to an efficient performance of biohythane production [24–26]. By doing so, this study aims (1) to continuously produce biohythane from HTL liquid products via two-stage fermentation using UASB and PBR; (2) to investigate the conversion pathways of HTL liquid products, especially the inhibitors in the two-stage and single-stage processes; (3) to compare the recoveries of energy and carbon between two-stage and single-stage fermentations through batch and continuous operation; and (4) to study the structure of the microbial community during biohydrogen and biomethane production based on Illumina MiSeq sequencing.

## Results and discussion

### Hydrothermal liquefaction of cornstalk

The yield of aqueous phase reached up to 39.3 ± 1.8% of the dry mass of cornstalk. The carbon and nitrogen balance showed that 30% of the carbon and 58% of the nitrogen were distributed in the aqueous phase. This result indicated that the aqueous phase from the HTL process was one key stage to recover carbon and nutrients. The HTL liquid products mostly consisted of reducing sugars, VFAs, furfural, and 5-HMF (Table 1), which occupied 93.3% of all products (based on the COD). Compared with previous results [23], a low yield of reducing sugars was observed, probably due to the reactor scale used in this study. Compared with the 500 mL reactor in a previous study, the bigger reactor (1.8 L) used in this study had a slower heating and cooling rate. The xylose and glucose produced through the hydrolysis of the hemicellulose and cellulose were over decomposed into acids [27]. The acids mainly consisted of acetic acid and lactic acid, which showed a similar distribution in the liquid products of hydrothermal liquefied beech wood [28]. The inhibitors furfural and 5-HMF were produced in the treatment, and the concentration of 5-HMF was higher than furfural. This result suggested that cellulose was degraded as the 5-HMF formed from its degradation [23], whereas the furfural which was produced from hemicellulose was converted to acids. The produced gas mainly consisted of carbon dioxide (99.7%) and hydrogen (0.03%). The heating value (HV) of the solid residues (21.7 MJ/kg) was significantly improved compared with that of raw cornstalk (15.4 MJ/kg). The solid residue mainly consisted of lignin, as the degradation of cellulose and hemicellulose reached 100 and 50%, respectively, at this hydrothermal treatment severity [23]. The solid residues could be used as solid fuels for combustion in a power plant [29].

**Table 1:** Characteristics of liquid products from HTL of lignocellulosic biomass

Items	This study^a^	Zhu et al. [23]	Yoshida et al. [28]
*Feedstock*	Cornstalk	Cornstalk	Beech wood
*HTL conditions* ^b^	260 °C, 20%	260 °C, 10%	380 °C, 3%
*Aqueous products*			
Reducing sugars (mg/L)	11.344 ± 3.011	5.991 ± 0.410	–
Total inorganic carbon (mg/L)	0.135 ± 0.002	–	–
Total organic carbon (mg/L)	28.600 ± 1.335	18.725 ± 1.033	–
Total nitrogen (mg/L)	1.045 ± 0.086	–	–
COD (mg/L)	76.192 ± 1.557	34.256 ± 0.880	–
Formic acid (mg/L)	8.509 ± 1.542	2.320 ± 0.560	0.100–0.900
Lactic acid (mg/L)	9.758 ± 1.392	4.830 ± 0.140	0.300–5.400
Acetic acid (mg/L)	22.336 ± 2.476	8.680 ± 0.740	6.600–13.500
Propionic acid (mg/L)	2.730 ± 0.856	7.280 ± 0.580	–
Butyric acid (mg/L)	9.072 ± 2.136	1.780 ± 0.370	–
5-HMF (mg/L)	1.350 ± 0.300	0.140 ± 0.010	0–3.700
Furfural (mg/L)	0.143 ± 0.042	1.850 ± 0.050	0.100–4.400

^a^a ± b represents the mean and standard deviation calculated from n ≥ 3

^b^Represents the HTL temperature X °C and total solid content Y%

### Operational performance of continuous anaerobic reactors

#### Two-stage biohythane systems

**Fig. 1:**
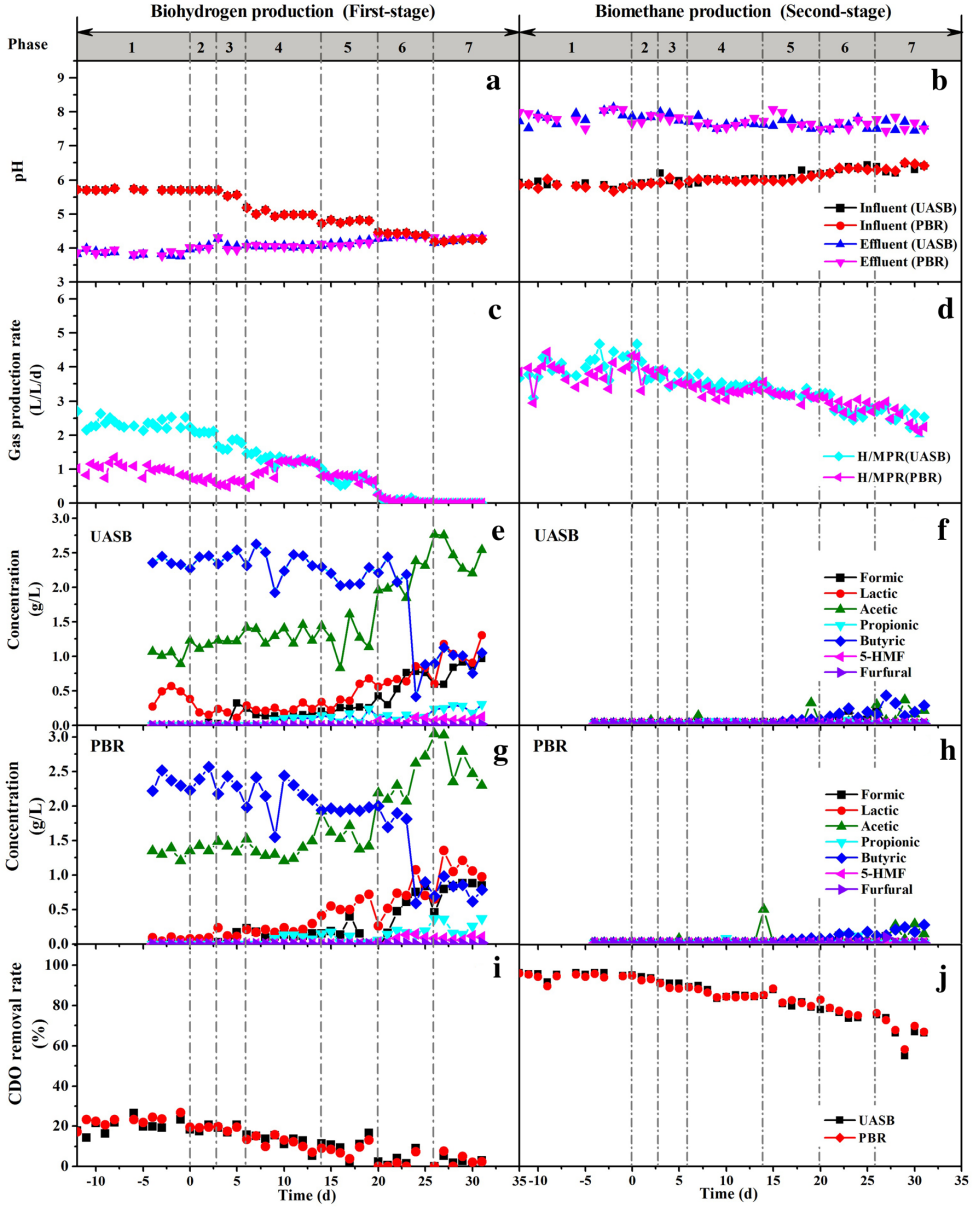
The changes of pH (**a**, **b**), gas production rate (**c**, **d**), concentrations of VFAs and furan derivatives (**e**–**h**), and COD removal (**i**, **j**) in two-stage process. The concentration of HTL liquid products was increased from 0 (*Phase 1*), 1 (*Phase 2*), 2 (*Phase 3*), 3 (*Phase 4*), 4 (*Phase 5*), 6 (*Phase 6*) to 8 g COD/L (*Phase 7*) in sequence to replace the synthetic wastewater. The minus time means the reactors were operated with synthetic wastewater only

The biohythane production setup consisted of two biohydrogen reactors (UASB-H, PBR-H) and biomethane reactors (UASB-M_1_, PBR-M_1_). The hydraulic retention time (HRT) for all reactors remained 12 h throughout all experiments unless elsewhere stated. A decrease of biohydrogen production in both UASB-H and PBR-H reactors was observed (Fig. 1). The hydrogen concentration in UASB-H was relatively stable (48.5 ± 8.5%) until the concentration of the HTL liquid products increased to 8 g COD/L (Additional file 1: Figure S1). However, the hydrogen concentration in PBR_H_ increased from 24.8% in Phase 1 to 41.2% in Phase 4 (Additional file 1: Figure S1). This result was caused by the decrease of the initial pH which suppressed the hydrogen-consuming reactions in PBR-H [30]. Biohydrogen production was undetectable when the concentration of the HTL liquid products reached 8 g COD/L (Phase 7). This result was probably caused by the low concentration of sugars in the HTL liquid products, which is the main resource for biohydrogen production during dark fermentation [31]. In addition, the high concentrations of acetic and butyric acid in the substrate were considered to be inhibitive for biohydrogen production [32]. What’s more, the initial concentration of 5-HMF and furfural in the feedstock increased as the loading of the HTL aqueous product increased. 5-HMF and furfural reached maximum values in Phase 7 as 142 and 15 mg/L, respectively. The presence of furan derivatives was reported to have a negative impact on biohydrogen production, which could lead to a metabolic shift from hydrogen-producing pathways (via acetate and butyrate) to non-hydrogen-producing pathways (via ethanol and lactate) [33]. The change of potential pathway was supported by the lactate concentration in the biohydrogen reactors in Phase 7 (Fig. 1e, g), which reached values of 1080 mg/L in UASB and 1126 mg/L in PBR, respectively. The values were slightly increased, compared with the influent concentration (1024 mg/L). 5-HMF was partially degraded in both UASB-H (41.4% of the initial quantity detected) and PBR-H (41.9% of the initial quantity detected) in Phase 7, whereas most of the furfural was degraded in UASB-H (85.0% of the initial quantity detected) and PBR-H (74.7% of the initial quantity detected). One study reported a similar finding with an initial concentration below 1 g/L during biohydrogen fermentation [21]. The result indicated that the biohydrogen process had the ability to degrade furfural and 5-HMF. COD removal in the biohydrogen reactors showed a similar change in the gas production trend. The COD removal rate in UASB-H decreased from 19.8% (Phase 1) to 2.2% (Phase 7). Similarly, this value also decreased from 21.9 to 2.8% in PBR-H.

The content of methane in UASB-M_1_ (73.9 ± 2.5%) and PBR-M_1_ (72.6 ± 3.6%) was relatively stable during operation (Additional file 1: Figure S1). The changes of pH before and after anaerobic digestion also supported a stable performance of methane production. Specifically, all the pH values of the effluents were above 7 (Fig. 1b), although the initial pH values of UASB-M1 and PBR-M1 were around 6, suggesting the acids in the HTL liquid products were converted to biogas (Fig. 1f, h). A slow decrease of the biomethane production rate was observed in the biohythane systems (Fig. 1). The methane production rates decreased to 2.53 (UASB-M_1_) and 2.54 (PBR-M_1_) L/L/day in Phase 7, respectively, corresponding to a decrease of the COD removal, at 67.2% in UASB-M_1_ and 68.6% in PBR-M_1_ (Phase 7), respectively. Most VFAs in the HTL liquid products were used for methane production, and the furfural and 5-HMF were undetectable in the effluent of the biomethane production reactors.

#### Single-stage biomethane systems

**Fig. 2:**
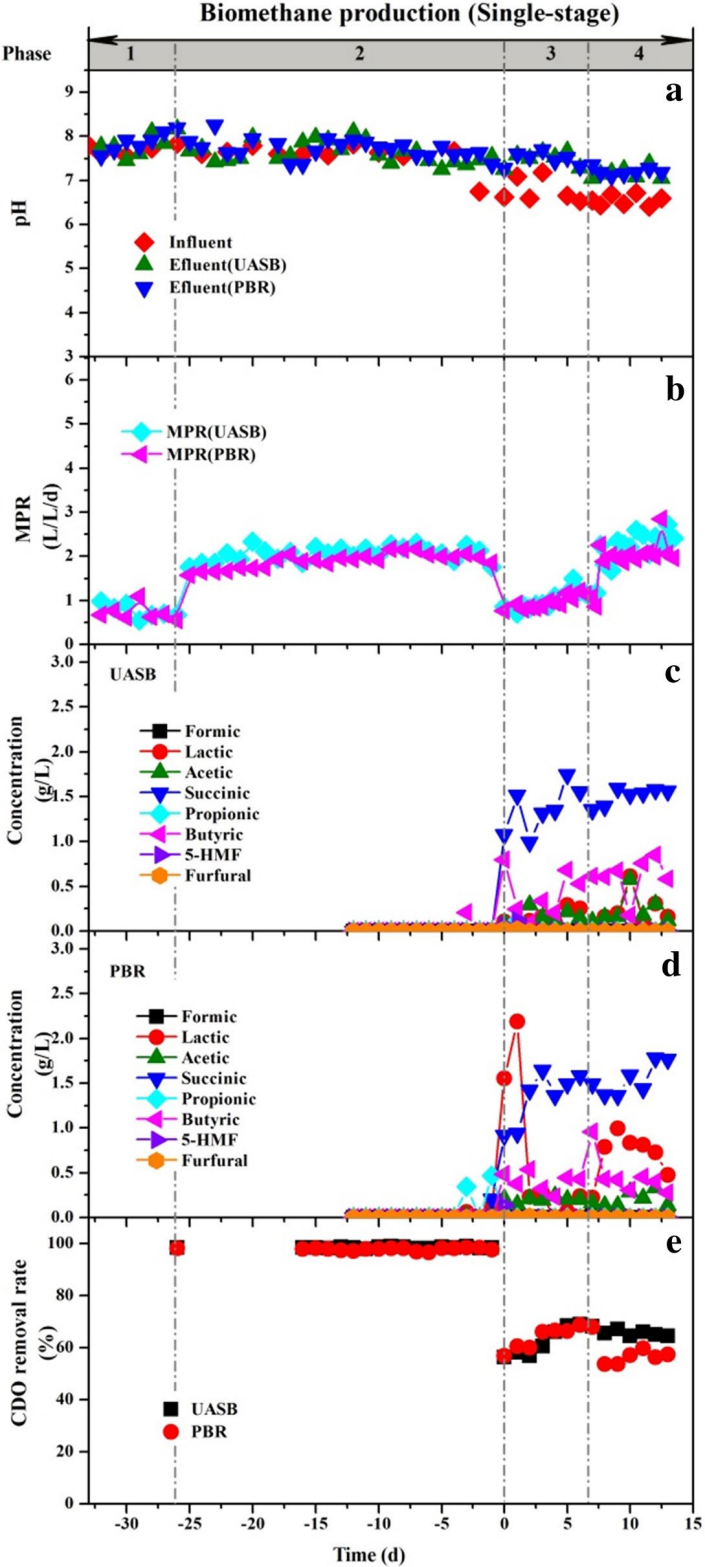
The changes of pH (**a**), gas production rate (**b**), VFAs concentrations (**c**, **d**), and COD removal rate (**e**) in single-stage systems. In *Phase 1* and *2*, the single-stage systems started with synthetic wastewater with a HRT of 48 and 24 h, respectively. In *Phase 3*, the HTL liquid products were used instead of synthetic wastewater with a HRT of 24 h. In *Phase 4*, the HRT was decreased to 12 h. The minus time means the reactors were operated with synthetic wastewater only

The single-stage systems for biomethane production (UASB-M_2_, PBR-M_2_) were set up using the same-scale reactors as the two-stage systems. The single-stage systems started with synthetic wastewater at Phase 1 and 2 with a HRT of 48 and 24 h, respectively. The total COD concentration of the influent was 8 g/L throughout all phases. The methane production in the single-stage systems showed a significant decrease when Phase 2 changed to Phase 3 (Fig. 2b), where the HTL liquid products were used instead of synthetic wastewater. By reducing the HRT from 24 to 12 h (Phase 4), methane production rates reached 2.27 in UASB-M_2_ and 2.07 L/L/day in PBR-M_2_. The COD removal decreased to 65.5 (UASB-M_2_) and 56.3% (PBR-M_2_), respectively. The methane content showed a stable performance in UASB-M_2_ (64.6 ± 4.9) and PBR-M_2_ (65.3 ± 4.1%) (Additional file 1: Figure S2). These values were lower than those in the methane reactors of the biohythane systems. Concentrations of furfural and 5-HMF were undetectable in the effluents, suggesting the complete degradation of these inhibitors in the single-stage system. The floating of granules was observed in the UASB-M2 in Phase 4 (Additional file 1: Figure S3). The floating granules accumulated around the gas–liquid–solid separator in the UASB, which probably resulted in the dysfunction of the separator and the wash out of the granules. The wash out of the granules caused by the presence of toxic compounds was also reported in a UASB used to treat the phenolic compounds [34]. However, the detailed reason for the floating of granules awaits further investigation.

In comparison, a better performance of methane production was achieved in the two-stage process. One possible reason was the degradation of fermentation inhibitors during biohydrogen production. Moreover, the biomethane process in the biohythane systems enhanced the acetogenesis process as previously reported [24]. The acetogenesis process is referred to as the rate-limiting procedure in the anaerobic fermentation of liquid products from HTL [35]. This assumption was verified by the differences between the metabolic products in the effluent of the single-stage and two-stage processes. Compared with the two-stage process, the effluent of the single-stage process had a higher concentration of VFAs, mainly consisting of butyric acid, lactic acid, and acetic acid (Figs. 1f, h, 2c, d). Specifically, the concentration of lactic acid in the effluent of the PBR-M_2_ systems reached 773 mg/L, whereas it was undetectable in the two-stage system. The results of the batch experiments exhibited similar results. In addition, a shorter lag phase (Additional file 1: Figure S4, Table S1), higher methane production (Additional file 1: Figure S4, Table S1), and COD removal (Additional file 1: Figure S5) were observed in the two-stage batch fermentation.

Table 2 shows the performance of biohythane production through the integration of the two-stage process and HTL, compared with other studies. The biohydrogen production was limited in this study, which was also observed when using heat-treated sunflower stalks and *Gelidium amansii* as the feedstock [33, 36]. These studies revealed that the fermentation inhibitors produced from hydrothermal products, including 5-HMF and furfural, were supposed to change the hydrogen-producing pathway to the non-hydrogen-producing pathway. However, a hydrogen yield of 212 mL/g sugar and 109.6 and 288 mL/COD was achieved using the liquid products from pretreated switchgrass [37], *Laminaria japonica* [38], and wheat straw [39], respectively. This was probably due to the various feedstock and treatment conditions (i.e., temperature, retention time, chemicals, and reactors) which resulted in different inhibitor concentrations. The further decomposition of the produced sugars to inhibitors should be avoided. Previous studies for the hydrothermal pretreatment of lignocellulosic biomass were mostly conducted in batch reactors (Table 2), where a low heating and cooling rate may have resulted in the decomposition of produced sugars during the heating or cooling process. A continuous treatment may curtail the production of inhibitors, as the timely separation of sugars could effectively avoid their continued decomposition. Ji et al. reported a high yield of reducing sugar ratio (60.80%) and a low content of furfural in a continuous reactor [40]. Hence, a better performance of biohydrogen production can be expected when glucose and xylose from lignocellulose are efficiently recovered under optimal HTL condition. The microbial community also plays an important role in the biogas production using HTL products. A high-rate reactor, which can retain a high density of microorganisms, seems to be more competitive. Kongjan et al. observed a higher hydrogen production rate in UASB and AF (anaerobic filter) reactor than conventional CSTR using the wheat straw hydrolysate from HTL treatment [39]. As for the biomethane production, Table 2 shows the HRT (0.5 day) utilized in this study was much lower than previous reports (25–65 days), and a higher COD removal and methane yield were observed.

**Table 2:** Comparison of integration of hydrothermal treatment and gas biofuels production in the literature and this study

Feedstock	Products	HTL process	Conditions	Fermentation process	HRT (day)	Gas yield	COD removal (%)	Reference
Algae	Methane	Batch (stainless steel cylinder), 100 °mL	0–1.5 h, 260–320 °C	Batch, 37 °C, Liquid products	62	–	44–61	Tommaso et al. [35]
Swine manure	Methane	–	–	Batch, 37 °C, Liquid products	65	~150–175 mL/g COD	45–55	Zhou et al. [58]
Sunflower stalks	Methane	Batch	30 min, 160 °C H_2_SO_4_	Batch, 37 °C, Mixture	45	278 mL/g VS		Hesami et al. [19]
Sugar beet pulp	Methane	Batch (thermostatic reactor), 600 mL	20 min, 160 °C	Batch, 37 °C, Mixture	25	502.5 mL/g VS		Ziemin´ski et al. [17]
Wheat straw	Methane	Batch (cylindrical steel tank), 6.2 L	1 h, 160 °C, NaOH	Batch, 35 °C, Mixture	31	224 mL/g TS		Sambusiti et al. [15]
Rice straw	Methane	Batch (hydrothermal reactor), 131 mL	10 min, 200 °C,	Batch, 35 °C, Mixture	60	132.7 mL/g VS		Chandra et al. [18]
Beech wood	Methane	Batch (Inconel-625 vessel), 5 mL	7–240 s, 380 °C	Batch, 50 °C, Liquid products	35	–	–	Yoshida et al. [28]
*Laminaria japonica*	Hydrogen	Batch (stainless steel vessel), 5 L	20 min, 170 °C	Batch, 35 °C, Mixture	3.5	109.6 mL/g COD		Jung et al. [38]
Wheat straw	Hydrogen	–	15 min, 180 °C	Continuous (CSTR, UASB AF), 70 °C, Liquid products	1–3	212 mL/g sugar		Kongjan et al. [39]
Sunflower stalks	Hydrogen	Batch (Stainless autoclave), 1 L	1 h, 170 °C, HCl	Batch, 35 °C, Liquid products	30	0	–	Monlau et al. [33]
*Gelidium amansii*	Hydrogen	Batch (high-pressure reactor) 30 L	15 min, 150 °C H_2_SO_4_	Batch, 35 °C, Liquid products	1.25	0	–	Parka et al. [36]
Switchgrass	Hydrogen	Batch (steam explosion reactor), 4 L	10 min, 190 °C	Continuous (UASB), 37 °C, Liquid products	0.42	288 mL/g COD	–	Veeravalli et al. [37]
Cornstalk	Hythane	Batch (High-pressure reactor), 1.8 L	0 min, 260 °C	Continuous(UASB, PBR), 37 °C, Liquid products	Hydrogen, 0.5 Methane, 0.5	H_2_, 0–146 mL/g COD CH_4_, 158–302 mL/g COD	H_2_, 19 − 2 CH_4_, 93 − 67	This study

**Fig. 3:**
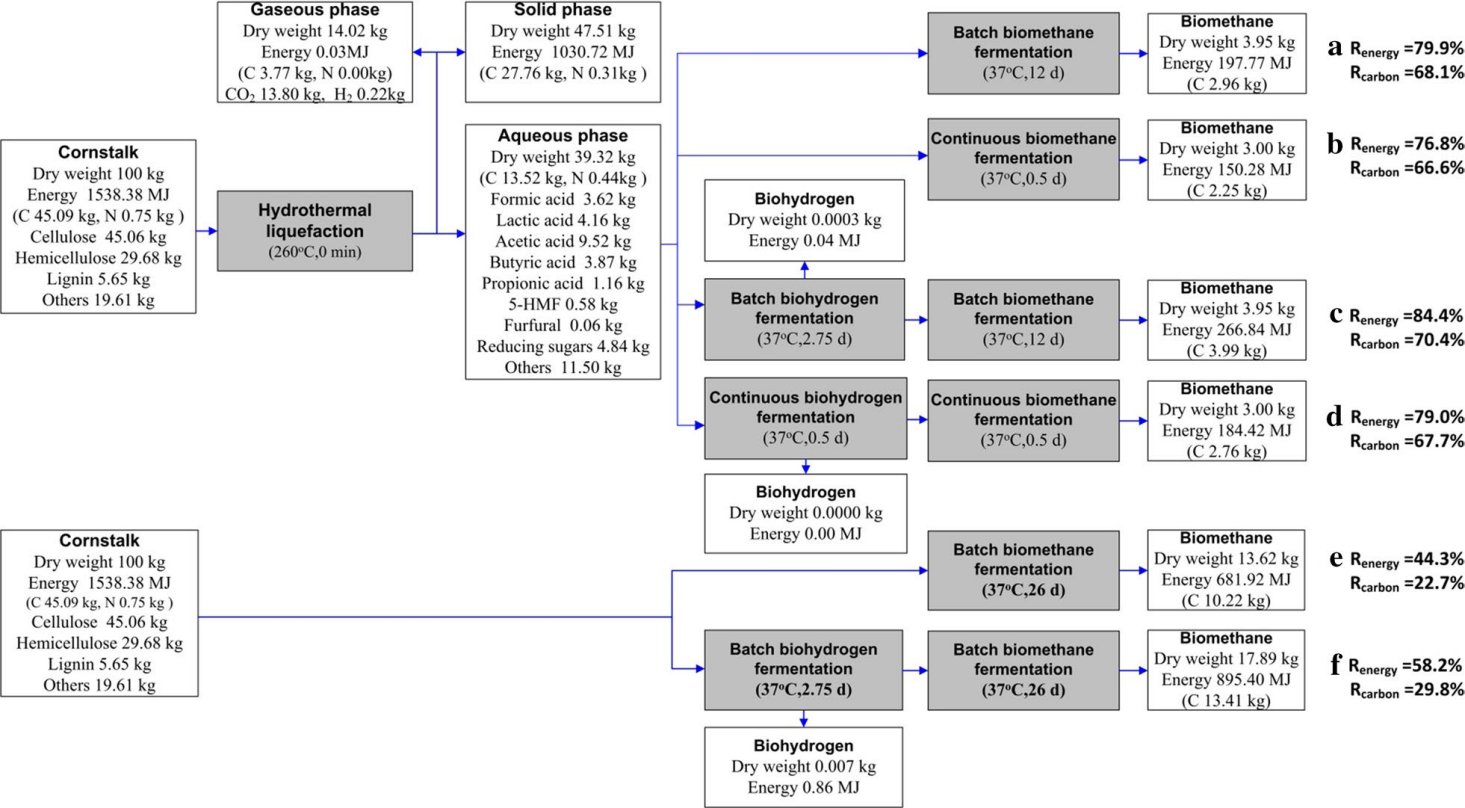
Carbon and energy recovery in the different conversion processes. **a** HTL and single-stage batch fermentation, **b** HTL and single-stage continuous fermentation, **c** HTL and two-stage batch fermentation, **d** HTL and two-stage continuous fermentation, **e** single-stage fermentation, and **f** two-stage fermentation

Figure 3 compares the carbon recovery (*R*_carbon_) and energy recovery (*R*_energy_) in different conversion processes. First, the integration of HTL and fermentation showed a higher carbon recovery (*R*_carbon_) and energy recovery (*R*_energy_) than direct fermentation of cornstalk. The integration of HTL and two-stage fermentation achieved a 79.0% *R*_carbon_ and 67.7% *R*_energy_ in continuous experiments. Batch experiments showed the potential of the HTL and two-stage fermentation, which could reach up to 84.4 and 79.0%, respectively. Second, *R*_carbon_ and *R*_energy_ in the two-stage process were improved in both the batch and continuous fermentation of cornstalk and HTL liquid products, compared to single-stage fermentation (Fig. 3). This result was also confirmed by a previous study [41]. However, in the integration of the HTL and fermentation process, the solid residue from HTL contributed to the largest fraction of the total *R*_carbon_ and *R*_energy_ (Fig. 3). Note that the integration of HTL and two-stage fermentation did not exhibit a higher *R*_carbon_ and *R*_energy_ than direct fermentation if only the biogas production part is considered. The solid residues with a high heating value could be used for energy production via combustion in a power plant [29]. In addition, the lignin-rich residue has also attracted increasing interest due to its potential to be utilized for value-added chemicals from the perspective of a biorefinery [42].

Although HTL is an energy intensive process, energy efficiency can be increased by recycling heat using a heat exchanger. One study reported that heat recovery could reach up to 90% [43]. The recycled heat could be used to preheat the feedstock or maintain the temperature of anaerobic reactors [44]. In addition, the dominant advantage of the current HTL and biohythane setup stems from its fast process (within 1 day) compared with direct fermentation of cornstalk (over 26 day) (Fig. 3). This process appears attractive for large-scale operation. The easily transportable HTL liquid products and the application of high-rate reactors would be helpful to improve the efficiency of biofuels production and reduce the capital investment and operating costs. However, detailed energy and economic analysis needs to be further evaluated.

### Microbial community analysis

**Fig. 4:**
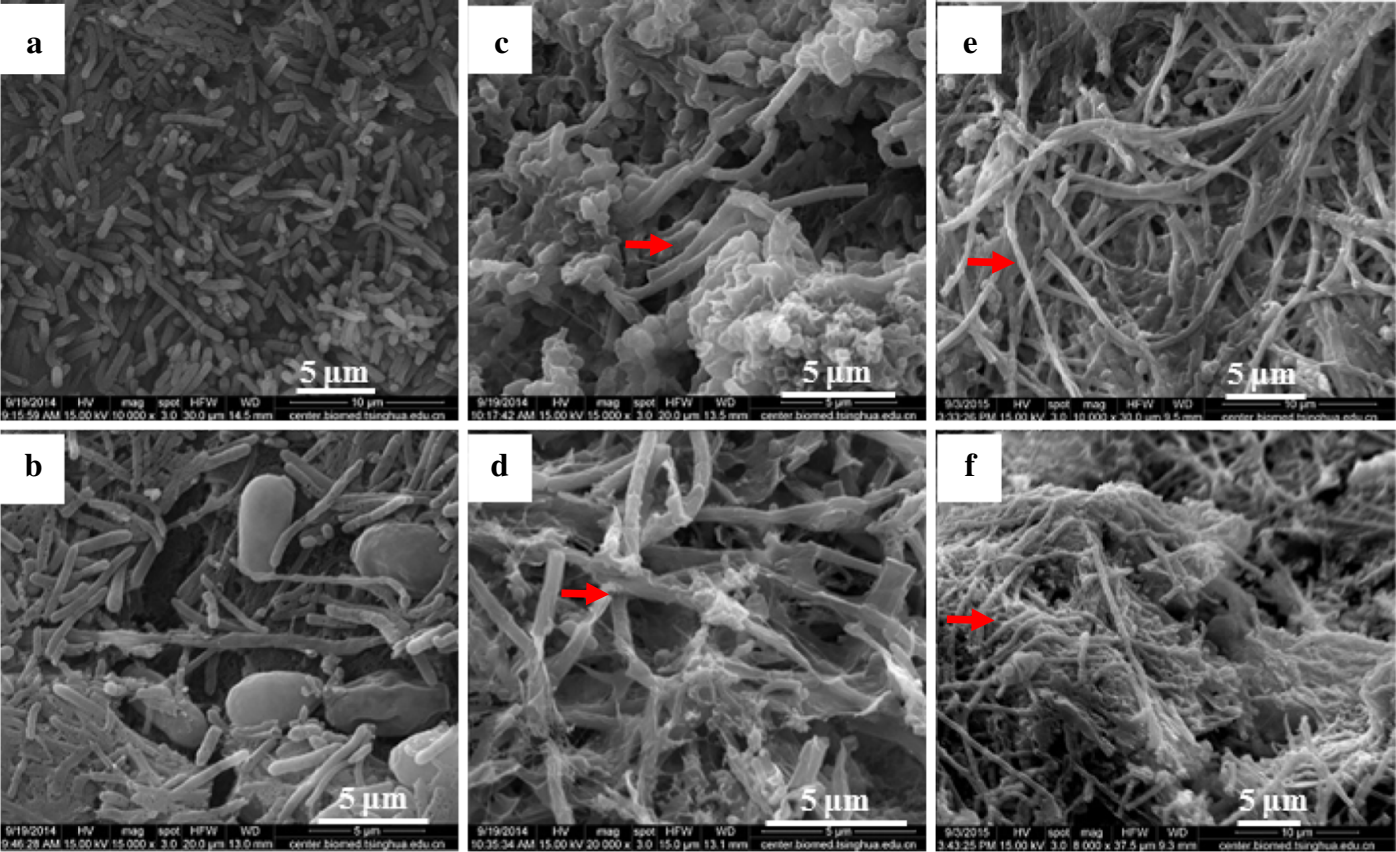
SEM images of microbial community of UASB_H_ (**a**) and PBR_H_ (**b**), UASB_M1_ (**c**), PBR_M1_ (**d**), UASB_M2_ (**e**), and PBR_M2_ (**f**). *Red arrows* indicate bamboo-like microbes

Scanning electron microscopy (SEM) images (Fig. 4) show the microbial morphology of granules and biofilms. As for the hydrogen reactors, the rod-shaped bacteria were dominant in both granules in UASB-H (Fig. 4a) and biofilms in PBR-H (Fig. 4b). However, a substantial amount of coccus-shaped bacteria were observed in the biofilms (Fig. 4b). As for the methane reactors, bamboo-like microorganisms were observed in the center of the methane-producing granules (Fig. 4c, e) and biofilms (Fig. 4d, f).

Illumina Miseq sequencing provided further analysis of the structure of the microbial community. Table 3 illustrates the differences in the microbial diversity. In the biohythane systems, the biohydrogen reactors (PBR-H, UASB-H) had a lower ACE, operational taxonomic units (OTUs), and Chao and Shannon indexes than the biomethane reactors. This result revealed the lower diversity of bacterial species in the biohydrogen process. Compared with PBR_M2_ and UASB_M2_, the lower ACE, OTUs, and Chao and Shannon indexes were observed in the PBR_M1_ and UASB_M1_, suggesting that the bacterial community of the methane reactors in the two-stage process had a lower diversity. However, the archaeal community showed a contrary result; the richness and diversity in the two-stage process were higher.

**Table 3:** Diversity analysis of microbial community for clustering at 97% identity

Sample	Bacterial community					Archaeal community				
	Chao	Shannon	Simpson	ACE	OTUs	Chao	Shannon	Simpson	ACE	OTUs
PBR_M1_	510	4.29	0.0447	503	486	21	1.16	0.5469	21	21
PBR_M2_	458	4.02	0.0384	449	408	24	0.37	0.8702	24	21
UASB_M1_	459	4.34	0.0297	459	446	21	1.65	0.3057	21	21
UASB_M2_	459	3.88	0.0832	453	416	33	0.96	0.5534	34	31
PBR_H_	253	2.45	0.1584	341	172	–	–	–	–	–
UASB_H_	70	1.38	0.3518	68	61	–	–	–	–	–

Further characterization of the microbial community is illustrated in Additional file 1: Figure S6. Significant differences in the microbial distribution between the biohydrogen and biomethane reactors in the two-stage process were observed (Additional file 1: Figure S6A). The biohydrogen reactors mainly consisted of the phylum *Firmicutes*, which occupied 99.5% in UASB-H and 94.8% in PBR-H, respectively. However, it occupied a much lower abundance in UASB-M_1_ (16.7%) and PBR-M_1_ (13.0%). The UASB-M_2_ and PBR-M_2_ had a higher abundance of *Firmicutes*, *Proteobacteria*, and *Chloroflexi* than UASB-M_1_ and PBR-M_1_. These bacteria were reported prevalent during the anaerobic degradation of aromatic organics, and were assumed relevant to the degradation of these inhibitors [45]. This analysis suggested that the aromatic organics in the HTL liquid products had been degraded in UASB_H_ and PBR_H_ before being fed into UASB-M_1_ and PBR-M_1_. Table 4 illustrates the potential metabolic role of microbes depending on their representative species in order to further understand their microbial function. The bacterial community for hydrogen production was mainly from the family *Clostridiaceae* (Additional file 1: Figure S6B). The family *Clostridiaceae* is responsible for biohydrogen production. In addition, the family *Clostridiaceae* may play a very important role in the conversion process of HTL liquid products. *Clostridiaceae* was observed during the production of biohydrogen and acetone–butanol–ethanol at high concentrations of furfural and 5-HMF [21, 46]. Efficient conversion of cellobiose to hydrogen was observed using *Clostridium* sp. R1 in the presence of toxic phenolic compounds (0–1500 mg/L) [47]. Another study proposed that the genus *Clostridium* was likely responsible for the conversion of phenol to benzoate, which was further degraded by acetogenesis bacteria [48]. The family *Bacillaceae* and *Pseudomonadaceae* were found to be related to the degradation of aromatic compounds as well (Table 4). Compared with UASB-M_1_ and PBR-M_1_, UASB-H and PBR-H showed a higher *Bacillaceae*, *Clostridiaceae*, *Leuconostocaceae*, *Planococcaceae*, *Pseudomonadaceae*, and *Streptococcaceae*. Most of them are related to acidogenesis which is an important stage for the production of biohydrogen (Table 4). The higher abundance of the family *Clostridiaceae*, *Bacillaceae*, and *Pseudomonadaceae* revealed a detoxification function in the biohydrogen production.

**Table 4:** Bacterial and archaeal families in the fermentation reactors

Families	Function	Taxonomy (phylum, class)	Metabolic features	Reference
*Acetobacteraceae*	Acidogenesis	*Proteobacteria, Alphaproteobacteria*	Ferment glucose and produce acetate	[59]
*Anaerolineaceae*	Acidogenesis Detoxification	*Chloroflexi, Anaerolineae*	Some species ferment glucose; major metabolic end products are VFAs and hydrogen; involved in phenol degradation	[60, 61]
*Bacillaceae*	Acidogenesis Detoxification	*Firmicutes, Bacilli*	Some species produce acid from carbohydrates; degrades polycyclic aromatic hydrocarbons	[62]
*Bacteriovoracaceae*	Unclear	*Proteobacteria, Deltaproteobacteria*	Invade the periplasm of their prey where they grow and replicate	[63]
*Caldilineaceae*	Acidogenesis	*Chloroflexi, Caldilineae*	Some species ferment glucose; major metabolic end products succinate, lactate, acetate, CO_2_ and traces of hydrogen	[64]
*Christensenellaceae*	Acidogenesis	*Firmicutes, Clostridia*	Some species ferment glucose; major metabolic end products are acetate and butyrate	[65]
*Comamonadaceae*	Acidogenesis Detoxification	*Proteobacteria, Betaproteobacteria*	Some species ferment pyruvate and glucose; degrade polycyclic aromatic compounds	[59]
*Clostridiaceae*	Acidogenesis Detoxification	*Firmicutes, Clostridia*	Some species ferment glucose; metabolic end products are hydrogen, butyrate, acetate and lactate; ferment methoxylated aromatics to acids, and degrade the aromatic amino acids	[62, 66, 67]
*Desulfovibrionaceae*	Acetogenesis Detoxification	*Proteobacteria, Deltaproteobacteria*	Some species utilize lactate and pyruvate; Major metabolic end products are acetate, hydrogen and CO_2_; degrade aromatic aldehydes and furfural	[50, 68]
*Geobacteraceae*	Detoxification	*Proteobacteria, Deltaproteobacteria*	Aromatic compounds are used by several species	[50]
*Lachnospiraceae*	Acidogenesis	*Firmicutes, Clostridia*	Some species ferment glucose; major metabolic end products are butyrate, succinate, acetate, lactate, formate and hydrogen	[69]
*Leuconostocaceae*	Acidogenesis	*Firmicutes, Bacilli*	Some species are heterofermentative and produce lactate	[69]
*Nitrospiraceae*	Unclear	*Nitrospirae, Nitrospira*	Some species consume for sulfate reduction	[53]
*Peptococcaceae*	Acidogenesis	*Firmicutes, Clostridia*	Fermentative, and syntrophy with hydrogenotrophs	[50, 62]
*Planococcaceae*	Acidogenesis	*Firmicutes, Bacilli*	Some species ferment glucose	[69]
*Porphyromonadaceae*	Acidogenesis	*Bacteroidetes, Bacteroidia*	Some species ferment glucose; end products of glucose fermentation are acetate, hydrogen, and CO_2_	[53]
*Propionibacteriaceae*	Acidogenesis	*Actinobacteria, Actinobacteria*	Ferment glucose; Main metabolic end products is propionic acid	[70]
*Pseudomonadaceae*	Detoxification	*Proteobacteria, Gammaproteobacteria*	Some spices degradation the aromatic compounds	[71]
*Ruminococcaceae*	Acidogenesis	*Firmicutes, Clostridia*	Ferment glucose; metabolic end products are hydrogen and VFAs	[21]
*Spirochaetaceae*	Acetate-oxidizing	*Spirochaetae, Spirochaetes*	Major metabolic end products are hydrogen and CO_2_	[51]
*Streptococcaceae*	Acidogenesis	*Firmicutes, Bacilli*	Carbohydrates are fermented to produce mainly lactic acid	[62]
*Synergistaceae*	Acidogenesis	*Synergistetes, Synergistia*	Some species ferment glucose and organic acids; Metabolic end products are acetate, CO_2_ and hydrogen; Co-culture with the hydrogenotrophic methanogens	[72, 73]
*Syntrophaceae*	Acetogenesis	*Proteobacteria, Deltaproteobacteria*	Propionate and butyrate-utilizing bacteria; Co-culture with hydrogenotrophic methanogens	[74, 75]
*Syntrophomonadaceae*	Acetogenesis	*Firmicutes, Clostridia*	Some species utilize fatty acids of 4-18 carbon atoms; Syntrophic association with hydrogenotrophic methanogens	[69, 76, 77]
*Syntrophorhabdaceae*	Acetogenesis Detoxification	*Proteobacteria, Deltaproteobacteria*	Syntrophic degradation of aromatic compounds, and produce acetate and hydrogen	[50]
*Thermotogaceae*	Acidogenesis	*Thermotogae, Thermotogae*	Able to ferment carbohydrates and peptides	[78]
*Methanobacteriaceae*	Methanogenic	*Euryarchaeota, Methanobacteria*	Hydrogenotrophic methanogens	[52]
*Methanoregulaceae*	Methanogenic	*Euryarchaeota, Methanomicrobia*	Hydrogenotrophic methanogens	[53]
*Methanosaetaceae*	Methanogenic	*Euryarchaeota, Methanomicrobia*	Acetoclastic methanogens	[52]
*Methanosarcinaceae*	Methanogenic	*Euryarchaeota, Methanomicrobia*	Hydrogenotrophic and acetoclastic methanogens	[52]
*Methanospirillaceae*	Methanogenic	*Euryarchaeota, Methanomicrobia*	Hydrogenotrophic methanogens	[52]

In terms of the biomethane reactors, the higher abundance of potential detoxification families including *Bacillaceae*, *Clostridiaceae*, *Geobacteraceae*, *Pseudomonadaceae*, and *Syntrophorhabdaceae* were found in UASB-M_2_ and PBR-M_2_. Syntrophic bacteria also have an important role in the degradation of inhibitors. The genus *Desulfovibrio* was observed in all methane reactors and was responsible for the degradation of furfural and aromatic compounds [49, 50]. The family *Syntrophorhabdaceae*, which could syntrophically degrade aromatic compounds, was also found to exist in all methane reactors. However, these families were found to have a low distribution in the biohydrogen reactors (Additional file 1: Figure S6), probably due to the separation of hydrogen and methane production in the biohythane systems. The family *Spirochaetaceae*, which functions as an acetate-oxidizing agent [51], was much higher in UASB-M_1_ (27.9%) and PBR-M_1_ (10.8%) than UASB-M_2_ (3.3%) and PBR-M_2_ (3.7%). This family of bacteria enhanced the conversion of acetate to methane and strengthened acetogenesis in the methane reactors in the two-stage systems.

In addition, the archaeal community in the methane reactors (Additional file 1: Figure S6D) mainly belonged to the family *Methanosaetaceae*, which is an acetotrophic methanogen [52]. Generally, UASB-M_2_ and PBR-M_2_ had a higher abundance of hydrogenotrophic methanogens. The hydrogenotrophic methanogens played an important role in the degradation of inhibitive compounds through oxidation by obligate syntrophs [53]. The produced hydrogen from obligate syntrophs needs be consumed, which otherwise would lead to the inhibition of the end product. This result corresponds to the higher distribution of syntrophic detoxification bacteria *Desulfovibrionaceae* and *Syntrophorhabdaceae* in the UASB-M_2_ and PBR-M_2_.

## Conclusion

This study demonstrated that biohythane production through two-stage fermentation was an attractive process for the recovery of energy and degradation of organic compounds from hydrothermal liquefied biomass. The energy and carbon recovery of the integrated HTL and continuous biohythane fermentation systems reached up to 79.0 and 67.7%, respectively. One critical challenge for biohythane production is the limited performance of hydrogen fermentation. Possible biological approaches to address this issue include the domestication of microbial consortium or upstream modification of metabolic pathways. Compared with the single-stage process, the two-stage process showed a more efficient gas production and COD removal. The two-stage process not only contributed to the improved quality of the gas fuels but also strengthened the biotransformation process due to the detoxifying function during biohydrogen production and stronger acetogenesis process during biomethane production. The higher detoxification bacteria in the biohydrogen process and the acetate-oxidizing bacteria in the biomethane process revealed by Illumina MiSeq sequencing supported the performance of gas biofuels production. The presented method might be a promising way to convert lignocellulosic biomass to biohythane.

## Methods

### Feedstock and HTL process

The cornstalk was collected from Golden Sun Farm (Beijing, China), and the content of cellulose, hemicellulose, and lignin in the cornstalk was 45.06 ± 0.70, 29.68 ± 0.31, and 5.65 ± 0.27%, respectively. HTL was conducted in a temperature controllable 1.8 L batch reactor (4578, Parr Instruments Co., Moline, IL, USA). In order to get an efficient degradation of cornstalk and recovery of reducing sugars and VFAs, the reaction temperature was set to 260 °C with a retention time of 0 min as previously described [23]. The reactor was purged with nitrogen gas twice to ensure oxygen-free conditions and to maintain an initial pressure of 2.5 MPa. The liquid products were achieved by vacuum filtration of the mixture after HTL. The HTL liquid products were diluted to desired concentrations with tap water before being fed to fermentation reactors.

### Biohythane and biomethane production

The UASB and PBR, which were made from transparent acrylic with a working volume of 2.5 L, were used to build up the fermentation systems. Carbon nanotubes (100 mg/L) were added into the UASB to accelerate granules formation, and polyethylene rings (1 cm diameter and 1 cm wide) were packed in the PBR. The reactors were maintained at 37 °C using a water jacket. The inoculum was obtained from the anaerobic reactor of the Xiaohongmen Municipal Wastewater Treatment Plant (Beijing, China). The inoculum of biohydrogen production was heat-pretreated before inoculation (100 °C, 15 min). The UASB and PBR were operated with synthetic wastewater before using the HTL liquid products. Regarding the synthetic wastewater, glucose was used as the carbon source, and NH_4_Cl served as the nitrogen source. The nutrients were added to the feedstock as previously described [54]. NaHCO_3_ was added at 0.5 g/g COD for the hydrogen reactors and 1 g/g COD for the methane reactors. The pH of the substrate for biohydrogen production was controlled by adding 20 mL of 2 mol/L HCl per 1 L substrate, whereas it was not controlled for biomethane production. The reactors were operated over 200 days to enrich the microorganisms. Two series of biohythane systems were established by sequentially connecting the biohydrogen and biomethane reactors [24]. One system consisted of two PBRs for biohydrogen and biomethane production in sequence, and the other system was composed of two UASB reactors. The concentration of HTL liquid products was increased from 0 to 8 g COD/L stepwise to replace the synthetic wastewater. Specifically, the concentration was 1 g COD/L at Phase 2, 2 g COD/L at Phase, 3 g COD/L at Phase 4, 4 g COD/L at Phase 5, 6 g COD/L at Phase 6, and 8 g COD/L at Phase 7, respectively.

The batch experiments were conducted using 250-mL glass flasks (200 mL working volume). The temperature was controlled at 37 °C by a water bath. The initial pH of biohydrogen production was controlled by adding HCl. Before biomethane production commenced, the pH was adjusted by adding 0.5 g NaHCO_3_. The gas was collected by gastight balloons, and the volume was measured by a syringe. All chemicals were of analytical grade and were purchased from Beijing Chemical Factory.

### Calculations

The accumulative production of biohydrogen and biomethane in the batch experiments was simulated by the modified Gompertz equation [35] (Eq. 1).

1 $$P = P_{\text{s}} \exp \left[ {-\exp \left( {{{R_{\text{m}} \times e} \mathord{\left/ {\vphantom {{R_{\text{m}} \times e} {P_{\text{s}} \times \left( {\lambda \text{ - }t} \right) + 1}}} \right. \kern-0pt} {P_{\text{s}} \times \left( {\lambda -t} \right) + 1}}} \right)} \right],$$

where *P* was the accumulative hydrogen or methane production (ml); *P*_s_ was the hydrogen or methane production potential (mL); *R*_m_ was the maximum hydrogen and methane production rate (mL/day); *e* is the exp(1) = 2.71828; *λ* was the lag time (day); and *t* was the incubation time (day).

During HTL, the yields of gases (*Y*_gases_, %), solid phase (*Y*_solid_, %), and the aqueous phase (*Y*_aqueous_, %) were calculated using Eqs. 2, 3, and 4, respectively.

2 $$Y_{\text{gases}} = \frac{{\varSigma X_{\text{a}} \times V/22.4 \times M_{\text{a}} /1000}}{{M_{\text{cornstalk}} }} \times 100{\% }$$

3 $$Y_{\text{solid}} = \frac{{M_{\text{solid}} }}{{M_{\text{cornstalk}} }} \times 100{\% }$$

4 $$Y_{\text{aqueous}} = 100{\% }-{\text{Y}}_{\text{gases}} - {\text{Y}}_{\text{solid}},$$

where *X*_a_ (%) and *M*_a_ (g/mol) are the volume concentration and molar mass of the gases (hydrogen, methane, or carbon dioxide), respectively. *V* is the total volume of the produced gas. *M*_cornstalk_ (kg) and *M*_solid_ (kg) are the dry mass of cornstalk and the solid phase after HTL, respectively.

The energy yield of hydrogen (*E*_H2_, MJ/kg cornstalk) was calculated as

5 $$E_{\text{H2}} = Y_{\text{H2}} /22.4 \times {\text{HV}}_{\text{H2}},$$

where *Y*_H2_ is the total hydrogen yield in HTL and fermentation (L/kg cornstalk), and HV_H2_ is the heating value of hydrogen (0.242 MJ/mol) [24].

The energy yield of methane (*E*_CH4_, MJ/kg cornstalk) was calculated as

6 $$E_{\text{CH4}} = Y_{\text{CH4}} /22.4 \times {\text{HV}}_{\text{CH4}},$$

where *Y*_CH4_ is the methane yield in HTL and fermentation (L/kg cornstalk), and HV_CH4_ is the heating value of methane (0.801 MJ/mol) [24].

The energy yield of the solid phase after HTL (*E*_solid_, MJ/kg cornstalk) was calculated as

7 $$E_{\text{solid}} = Y_{\text{solid}} \times HV_{\text{solid}},$$

where HV_solid_ is the heating value of solid phase (MJ/kg).

The energy recovery (*R*_energy_, %) was proposed to represent the ratio of produced energy to chemical energy of the feedstock. The produced energy included energy from the solid phase after HTL (*E*_solid_), biohydrogen (*E*_H2_), and biomethane (*E*_CH4_). The energy recovery can be calculated as

8 $$R_{\text{energy}} = \frac{{E_{\text{solid}} + E_{\text{H2}} + E_{\text{CH4}} }}{{{\text{HV}}_{\text{cornstalk}} }} \times 100{\% },$$

where HV_corstalk_ is the heating value of cornstalk (MJ/kg).

The heating values of the solid (HV_solid_, MJ/kg) and corn straw (HV_cornstalk_, MJ/kg) were calculated according to the Dulong formula [55]:

9 $${\text{HV}} = 0.3383C + 1.422(H-O/8),$$

where *C*, *H*, and *O* are the mass percentages of carbon, hydrogen, and oxygen, respectively.

The carbon recovery (*R*_carbon_, %) was proposed to represent the ratio of carbon in the energy products (biomethane and solid phase) to the carbon in the feedstock. The carbon recovery can be calculated as

10 $$R_{\text{carbon}} = \frac{{Y_{\text{solid}} \times C_{\text{solid}} + Y_{\text{CH4}} /22.4 \times 44/1000 \times C_{\text{CH4}} }}{{C_{\text{cornstalk}} }} \times 100{\% },$$

where *C*_cornstalk_, *C*_solid_, and *C*_CH4_ are the carbon content (%) in the cornstalk, solid phase, and biomethane, respectively.

### Analytical methods

Gas volume was monitored using gas meters at room temperature (25 ± 3 °C) and corrected under standard condition (273.15 K, 101.325 kPa). The gas content, including hydrogen, methane, and carbon dioxide, was determined by a gas chromatography (GC1490, Agilent Technologies, USA) equipped with a thermal conductivity detector and a stainless steel column packed with TDX-01. Nitrogen was used as the carrying gas at a flowrate of 50 mL/min. The temperature of the injector, column, and detector was 150, 120, and 150 °C, respectively. The acids, furfural, and 5-HMF were analyzed by high-performance liquid chromatography (10A, Shimadzu, Japan) equipped with an ultraviolet detector and a synergi 4u Hydro-RP (Phenomenex) column. 5 mmol/L H_2_SO_4_ was used as the mobile phase at a flowrate of 1 mL/min, and the oven temperature was 40° C. Reducing sugars were determined by the 3,5-dinitrosalicylic acid method as previously described [23]. Element components of the cornstalk and solid residues were analyzed using a CHN analyzer (CE-440 Elemental Analyzer, Exeter Analytical, Inc. USA). The microbial morphology was observed by SEM (Quanta 200, FEI, USA) as previously described [56]. The phylogenetic diversity of the microbial consortium was analyzed via Illumina MiSeq sequencing. Primers 515F (5′-barcode-GTGCCAGCMGCCGCGG-3′) and 907R (5′-CCGTCAATTCMTTTRAGTTT-3′) for bacteria were used [54]. Primers Arch344F (5′-ACGGGGYGCAGCAGGCGCGA-3′) and Arch915R (5′-GTGCTCCCCCGCCAATTCCT-3′) for archaea were used [57]. The PCR process was conducted as previously described [54]. Amplicons were extracted from 2% agarose gels, purified using the AxyPrep DNA gel extraction kit (Axygen Biosciences, USA), and quantified using QuantiFluor ST (Promega, USA). The purified amplicons were pooled in equimolar and paired-end sequenced on an Illumina MiSeq platform. The raw reads were deposited into the National Center for Biotechnology Information (NCBI) Sequence Read Archive (SRA) database. The raw fastq files were demultiplexed and quality-filtered using Quantitative Insights into Microbial Ecology (QIIME). The phylogenetic affiliation of each 16S rRNA gene sequence was analyzed by a RDP Classifier (http://rdp.cme.msu.edu/) against the silva (SSU115) 16S rRNA database using a confidence threshold of 70%.

## Additional file

**Additional file 1: Figure S1.** Gas content in the two-stage fermentation. **Figure S2.** Gas content in the single-stage fermentation. **Figure S3** The floating of granules in the UASB biomethane systems. **Figure S4** Accumulative biohydrogen production (A), hydrogen content (B), methane production (C), methane content (D) in two-stage process, and accumulative methane production (E), methane content (F) in single-stage process. **Figure S5** COD removal in the two-stage and single-stage batch fermentation. **Figure S6** Taxonomic classification of microbial community in biohythane and biomethane systems at the phylum (A, C) and family (B, D) levels through Illumina Miseq sequencing. **Table S1** The biogas yield (*Pm*), maximum production rate(*Rm*), lag phase (*λ*), and (R^2^) in the batch fermentation.

## Abbreviations

HTL: hydrothermal liquefaction
OLR: organic loading rate
UASB: upflow anaerobic sludge blanket
PBR: packed bed reactor
CSTR: continuous stirred anaerobic reactors
AF: anaerobic filter
5-HMF: 5-hydroxymethyl furfural
SEM: scanning electron microscopy
TN: total nitrogen
VFAs: volatile fatty acids
COD: chemical oxygen demand
HPR: hydrogen production rate (L/L/day)
MPR: methane production rate (L/L/day)
HRT: hydraulic retention time
UASB-H: upflow anaerobic sludge blanket in two-stage system for hydrogen production
PBR-H: packed bed reactor in two-stage system for hydrogen production
UASB-M_1_: upflow anaerobic sludge blanket in two-stage system for methane production
PBR-M_1_: upflow anaerobic sludge blanket in two-stage system for methane production
UASB-M_2_: upflow anaerobic sludge blanket in single-stage system for methane production
PBR-M_2_: upflow anaerobic sludge blanket in single-stage system for methane production
NCBI: National Center for Biotechnology
SRA: information sequence read archive
QIIME: quantitative insights into microbial ecology
TS: total solids
VS: volatile solids
