# Erratum to: Continuous production of biohythane from hydrothermal liquefied cornstalk biomass via two-stage high-rate anaerobic reactors

**DOI:** 10.1186/s13068-017-0716-1

**Published:** 2017-02-03

**Authors:** Bu-Chun Si, Jia-Ming Li, Zhang-Bing Zhu, Yuan-Hui Zhang, Jian-Wen Lu, Rui-Xia Shen, Chong Zhang, Xin-Hui Xing, Zhidan Liu

**Affiliations:** 1 Laboratory of Environment-Enhancing Energy (E2E), and Key Laboratory of Agricultural Engineering in Structure and Environment, Ministry of Agriculture, College of Water Resources and Civil Engineering, China Agricultural University, Beijing, 100083 China; 2 Department of Agricultural and Biological Engineering, University of Illinois at Urbana-Champaign, Urbana, 61801 USA; 3 Key Laboratory of Industrial Biocatalysis of Ministry of Education of China, Beijing, 100084 China; 4 Department of Chemical Engineering, Institute of Biochemical Engineering, Tsinghua University, Beijing, 100084 China

## Erratum to: Biotechnol Biofuels (2016) 9:254 DOI 10.1186/s13068-016-0666-z

After this article [1] was published, the authors realised that the funding number for The National Natural Science Foundation of China was incorrect. The correct funding number is as follows: 51561145013, U1562107.
