# Supplementary material for: Continuous production of biohythane from hydrothermal liquefied cornstalk biomass via two-stage high-rate anaerobic reactors
Source: Biotechnol Biofuels. 2016 Nov 21;9:254. doi: 10.1186/s13068-016-0666-z (PMC5117538; doi:10.1186/s13068-016-0666-z)
Supplement: Supplementary file 1 — Additional file 1: Figure S1. Gas content in the two-stage fermentation. Figure S2. Gas content in the single-stage fermentation. Figure S3 The floating of granules in the UASB biomethane systems. Figure S4 Accumulative biohydrogen production (A), hydrogen content (B), methane production (C), methane content (D) in two-stage process, and accumulative methane production (E), methane content (F) in single-stage process. Figure S5 COD removal in the two-stage and single-stage batch fermentation. Figure S6 Taxonomic classification of microbial community in biohythane and biomethane systems at the phylum (A, C) and family (B, D) levels through Illumina Miseq sequencing. Table S1 The biogas yield (Pm), maximum production rate(Rm), lag phase (λ), and (R2) in the batch fermentation. [file 13068_2016_666_MOESM1_ESM.docx]

Additional file 1

**Continuous production of biohythane from hydrothermal liquefied cornstalk biomass via two-stage high-rate anaerobic reactors**

Bu-Chun Si^1^, Jia-Ming Li^1^, Zhang-Bing Zhu^1^, Yuan-Hui Zhang^2^, Jian-Wen Lu^1^, Rui-Xia Shen^1^, Chong Zhang^3,4^, Xin-Hui Xing^3,4^, Zhidan Liu^1*^

1 Laboratory of Environment-Enhancing Energy (E2E), and Key Laboratory of Agricultural Engineering in Structure and Environment, Ministry of Agriculture, College of Water Resources and Civil Engineering, China Agricultural University, Beijing 100083, China

2 Department of Agricultural and Biological Engineering, University of Illinois at Urbana-Champaign, Urbana 61801, America

3 Key Laboratory of Industrial Biocatalysis of Ministry of Education of China, Beijing, 100084, China

4 Institute of Biochemical Engineering, Department of Chemical Engineering, Tsinghua University, Beijing, 100084, China

*Corresponding author: zdliu@cau.edu.cn; Fax: +86-10-62737329; Tel.: +86-10-62737329.

E-mail address: Bu-Chun Si (sibuchun@cau.edu.cn); Jia-Ming Li (jmlee@cau.edu.cn); Zhang-Bing Zhu (zhuzhangbing@163.com); Yuan-Hui Zhang (yzhang1@illinois.edu); Jian-Wen Lu (jianwenlu2012@126.com); Rui-Xia Shen (shenruixia@ cau.edu.cn); Chong Zhang (chongzhang@mail.thu.edu.cn); Xin-Hui Xing (xhxing@mail.tsinghua.edu.cn); Zhidan Liu (zdliu@cau.edu.cn)

**Fig. S1** Gas content in the two-stage fermentation.

**Fig. S2** Gas content in the single-stage fermentation**.**

**Fig. S3** The floating of granules in the UASB biomethane systems.

**Fig. S4** Accumulative biohydrogen production (A), hydrogen content (B), methane production (C), methane content (D) in two-stage process, and accumulative methane production (E), methane content (F) in single-stage process.

**Fig. S5** COD removal in the two-stage and single-stage batch fermentation.

**Fig.S6** Taxonomic classification of microbial community in biohythane and biomethane systems at the phylum (A, C) and family (B, D) levels through Illumina Miseq sequencing.

**Table S1** The biogas yield (*Pm*), maximum production rate(*Rm*), lag phase (*λ*), and (R^2^) in the batch fermentation

**Fig. S1** Gas content in the two-stage fermentation.





**Fig. S2** Gas content in the single-stage fermentation.





**Fig.S3** The floating of granules in the UASB biomethane systems


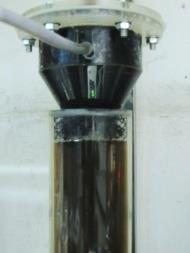


**Fig. S4** Accumulative biohydrogen production (A), hydrogen content (B), methane production (C), methane content (D) in two-stage process, and accumulative methane production (E), methane content (F) in single-stage process. HM3, HM2, HM1, and HM0 were two-stage fermentation of the mixture of HTL liquid products and glucose. Their ratios of HTL liquid products and glucose were 1:0, 1:1, 1: 3 and 0:1, respectively. M was the single-stage fermentation of HTL liquid products. C1 and C2 were single-stage and two-stage fermentation of cornstalk, respectively.


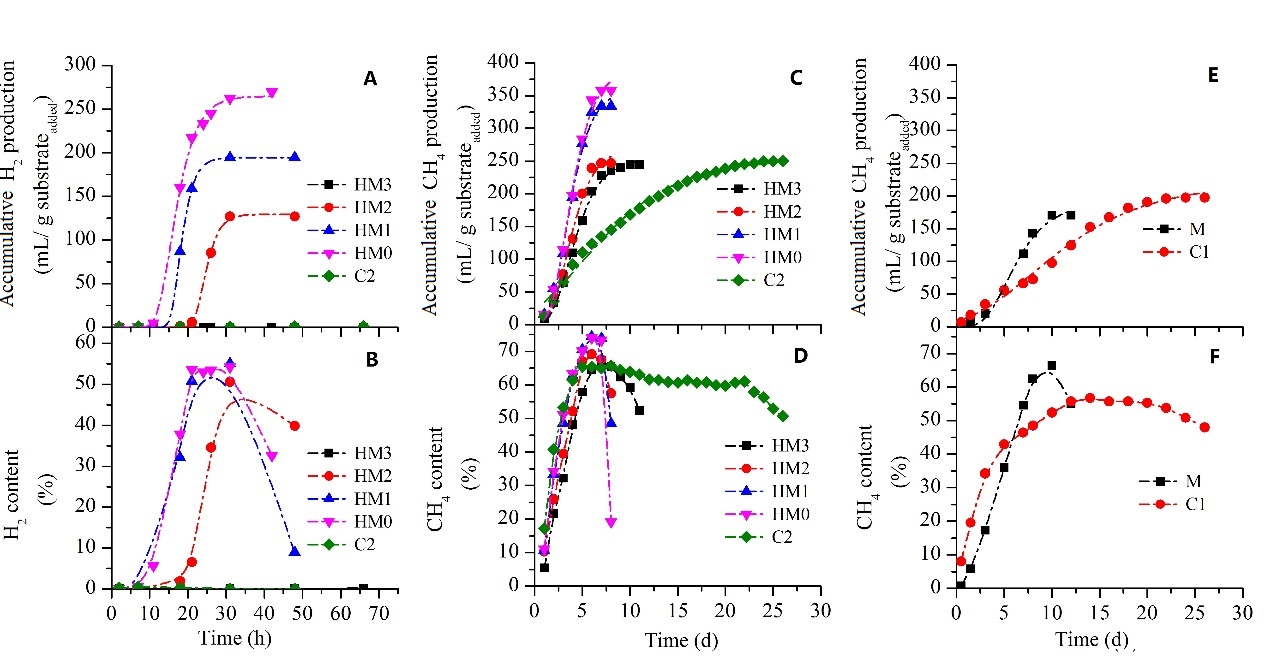


**Fig. S5** COD removal in the two-stage and single-stage batch fermentation. HM3, HM2, HM1, and HM0 were two-stage fermentation of the mixture of HTL liquid products and glucose. Their ratios of HTL liquid products and glucose were 1:0, 1:1, 1: 3 and 0:1, respectively. M was the single-stage fermentation of HTL liquid products.


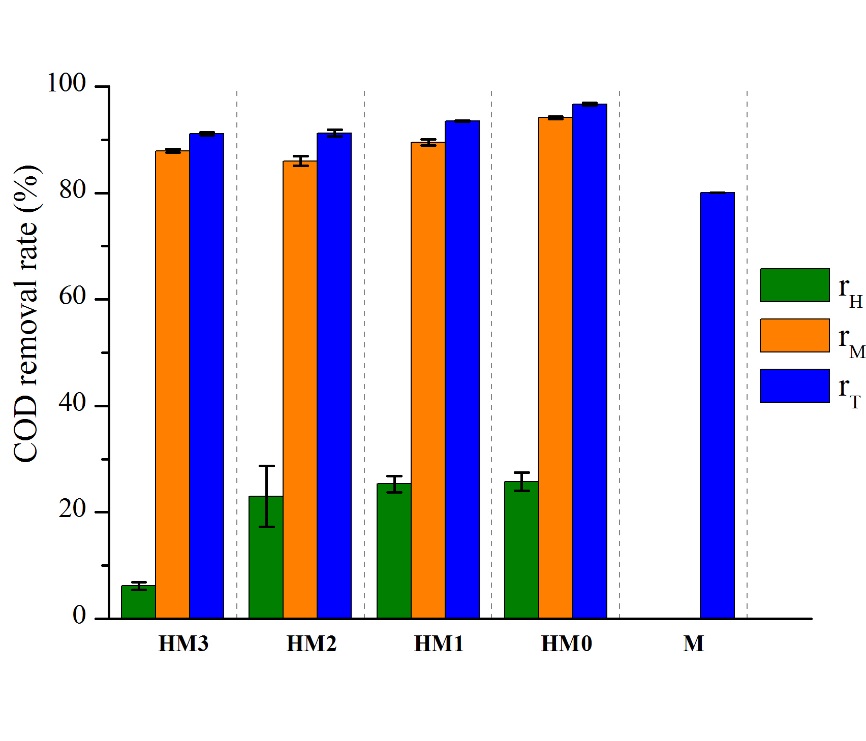


**Fig. S6** Taxonomic classification of microbial community in biohythane and biomethane systems at the phylum (A, C) and family (B, D) levels through Illumina Miseq sequencing.


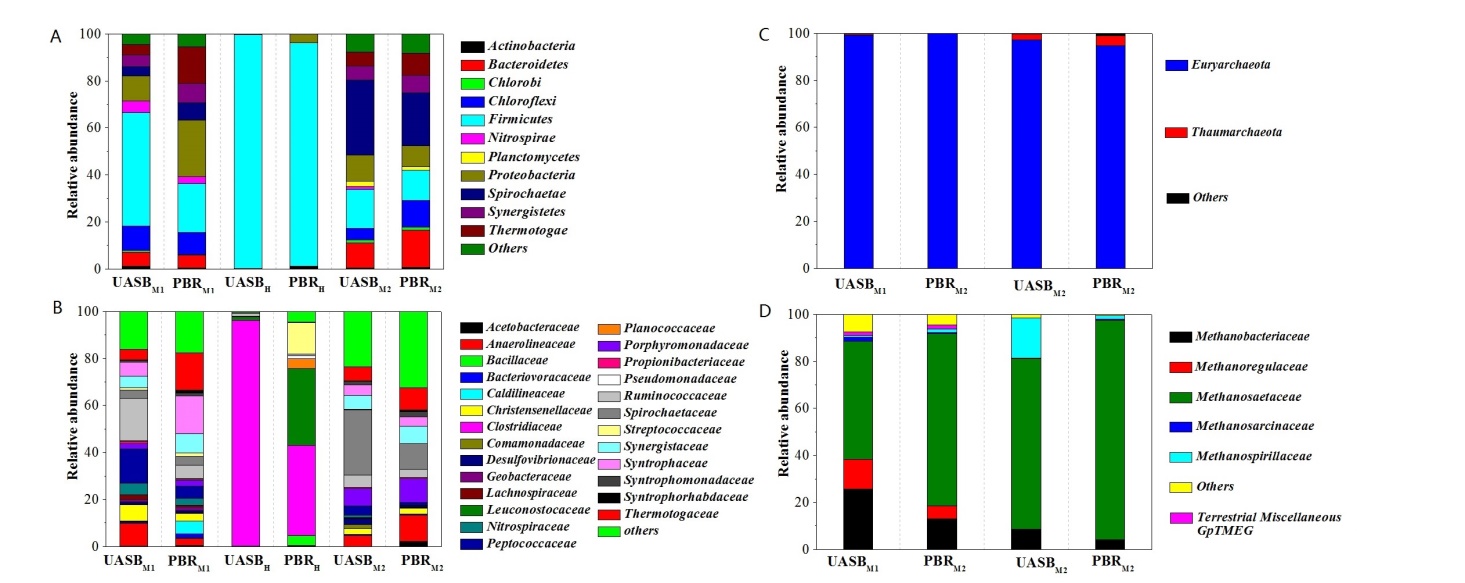


**Table S1** The biogas yield (*Pm*), maximum production rate (*Rm*), lag phase (*λ*), and (*R^2^*)in the batch fermentation. HM3, HM2, HM1, and HM0 were two-stage fermentation of the mixture of HTL liquid products and glucose. Their ratios of HTL liquid products and glucose were 1:0, 1:1, 1: 3 and 0:1, respectively. M was the single-stage fermentation of HTL liquid products. C1 and C2 were single-stage and two-stage fermentation of cornstalk, respectively

|  | *Two-stage (biohydrogen production)* | | | | |  | *Two-stage (biomethane production)* | | | | |  | *Single-stage biomethane production* | |
| --- | --- | --- | --- | --- | --- | --- | --- | --- | --- | --- | --- | --- | --- | --- |
|  | HM3 | HM2 | HM1 | HM0 | C2 |  | HM3 | HM2 | HM1 | HM0 | C2 |  | M | C1 |
| *Pm* ^a^( mL/ g substrate_added_) | 0.1 | 127.0 | 194.3 | 269.8 | 0.8 |  | 244.7 | 246.3 | 333.9 | 358.3 | 250.4 |  | 170.3 | 190.7 |
| *Rm* ^b^( mL/d) | - | 490.8 | 794.4 | 640.8 | - |  | 51.6 | 61.4 | 87.2 | 89.4 | 18.4 |  | 29.0 | 12.1 |
| *λ* ^b^ (d) | - | 0.90 | 0.64 | 0.49 | - |  | 1.73 | 1.64 | 1.63 | 1.64 | 0 |  | 2.91 | 1.24 |
| *R^2^* | - | 0.9987 | 0.9999 | 0.9983 | - |  | 0.9946 | 0.9843 | 0.9898 | 0.9914 | 0.9886 |  | 0.9901 | 0.9913 |

^a^ values from the experiments value, ^b^ values from the modified Gompertz equation
